# Editorial: Cognitive Development in Informal Learning Institutions: Collaborations Advancing Research and Practice

**DOI:** 10.3389/fpsyg.2021.827361

**Published:** 2022-01-17

**Authors:** Catherine A. Haden, Janet J. Boseovski, Thanujeni Pathman

**Affiliations:** ^1^Department of Psychology, Loyola University Chicago, Chicago, IL, United States; ^2^Department of Psychology, University of North Carolina at Greensboro, Greensboro, NC, United States; ^3^Department of Psychology, York University, Toronto, ON, Canada

**Keywords:** informal learning, naturalistic, cognitive development, museums, collaborative

## Overview

Researchers in cognitive development and developmental science more broadly are encouraged to bring our science into the “messiness of the real world” (Golinkoff et al., 2017, p. 1407). Many have heeded this call by establishing partnerships with community and educational institutions (e.g., museums, science centers, libraries), and in some cases, engaging in research that can serve real-world applications (Callanan, 2012; Sobel and Jipson, 2016; Haden, 2020). The goal of this Research Topic was to spotlight the growing number of collaborations with informal learning institutions and illustrate the cutting-edge cognitive and social-cognitive research occurring through these partnerships across a range of topics.

We thank all authors who submitted manuscripts and reviewers that provided thoughtful critiques. Their work resulted in nine empirical articles and one perspective article that successfully met our Research Topic criteria.

## Contributions

Several articles in this collection point to how the deliberate design of museum exhibits and facilitation strategies used by practitioners can support parent-child conversations and children's learning. Letourneau et al. considered how staff facilitation vs. labels printed on exhibits affected whether and how children and caregivers explored a science exhibit. They found little difference in families' goal setting across the facilitation and exhibit label conditions, but prompts by staff led caregivers to be more hands-off, whereas exhibit labels seemed to encourage more actions between caregivers and children during the science activity. Marcus et al. used design-based research methods to understand how iterations of a program at a children's museum promoted engineering engagement and talk. Design features that promoted testing best encouraged family engagement in engineering practices when coupled with facilitation by museum staff members. Shivaram et al. considered whether the presence and types of signage in a food pantry promoted learning opportunities for children. They showed that the presence of signs increased the number of conversations between caregivers and children, but only academically-relevant signs engendered high-quality conversations about key learning objectives. Tõugu found that families receiving a worksheet with a prompt to experiment during a museum-based science-related activity about shadows engaged in more experimentation than those not receiving the prompt. However, families receiving the experimentation activity prompt also had children who provided fewer explanations, leading Tõugu to suggest that prompts offered *via* worksheets might actually distract from deep engagement with hands-on exhibits. Joy et al. considered, for example, how children's behaviors in an exhibit varied according to whether it was interactive, showing that interactive exhibits promoted engagement but that non-interactive exhibits prompted more scientific explanations from children. Jee and Anggoro offer ideas about how research on relational thinking can be leveraged through exhibit design to make the comparison of natural specimens and scientific models salient.

Another common thread connecting across several articles in this collection is that storytelling and narratives can reveal and further advance children's learning from experiences in informal educational settings. Callanan et al. used a hands-on animation exhibit to encourage family storytelling during an exhibition experience, finding that families who told stories also engaged in more explanatory science discussions in other areas of the exhibition. Marcus et al. prompted children's narrative reflections immediately after an exhibit experience in a children's museum to gauge how variations in the exhibit program led to different amounts of talk about engineering. Attisano et al. found that older children in their study recalled more about a machine, and seemed more knowledgeable, compared to younger children. Prompts provided to some of the children to focus on the internal parts of the machine did not end up affecting children's talk and learning. Kian et al. analyzed children's autobiographical event memory and knowledge retention following a week-long summer camp at a zoo, finding that age-related differences varied based on the type of information being recounted and whether the experience had more or less self-relevance for the child. Finally, Marble et al. studied how children judged information about animals from experts and non-experts, revealing that children believed positive rather than negative statements about an animal irrespective of expertise, but remembered neutral information best.

## Future Directions

This collection signals important future directions in research and practice for the field. We highlight two here, but also refer readers to the discussions offered by authors of this collection.

First, the work in this collection points to everyday contexts and practices that can advance children's understanding, and skills in various academic areas. But what we see less of in this collection, and indeed field-wide, is how families from different cultural and social backgrounds learn in informal educational settings, as well as design efforts to support that learning. We need to take further crucial steps to co-develop and co-implement design and facilitation strategies in collaboration with diverse community members, to create informal educational opportunities that better reflect culturally relevant ways of teaching and learning, while being mindful not to promote erroneous deficit viewpoints (see Solis and Callanan, 2016).

The second area of challenge is to understand the conditions that can foster learning transfer across informal settings (e.g., museum to home) and across informal and formal educational settings (e.g., museum to school). Several of the articles in this collection point to how opportunities to reflect on informal experiences can reveal learning, and that chances to tell stories and recount experiences can be important in the process of bridging across learning experiences. Indeed, a critical factor in successful learning and transfer is whether the knowledge that is gained in one setting can be represented or re-represented so that it can be accessed in another context over time (e.g., Bransford and Schwartz, 1999; Jant et al., 2014). Learning transfer is something that many practitioners seek; they want their work to accrue benefits long after, for example, a museum visit is complete. Future work on storytelling and remembering informal learning experiences can help to realize learning transfer. Additionally, there is a paucity of research in which the same families are studied across different informal learning experiences and contexts. Undertaking such work could greatly inform both research and informal learning practice.

## Author Contributions

TP wrote an initial draft of the editorial which was then edited by all authors. The topic was initiated by TP. TP invited JB and CH to join as co-editors. All authors contributed equally to the proposal of the Research Topic, managed submissions in consultation with each other, and contributed equally to the writing of this editorial.

## Conflict of Interest

The authors declare that the research was conducted in the absence of any commercial or financial relationships that could be construed as a potential conflict of interest.

## Publisher's Note

All claims expressed in this article are solely those of the authors and do not necessarily represent those of their affiliated organizations, or those of the publisher, the editors and the reviewers. Any product that may be evaluated in this article, or claim that may be made by its manufacturer, is not guaranteed or endorsed by the publisher.
